# Psychological support for people affected by scandals caused by serious and sustained failings of statutory services and government: lessons from the infected blood scandal and Infected Blood Inquiry

**DOI:** 10.1192/bjo.2025.10901

**Published:** 2025-11-25

**Authors:** Jessica Carlisle, Eva Cyhlarova, Emily Warren, Martin Knapp, Ellen Nolte

**Affiliations:** Care Policy and Evaluation Centre, London School of Economics and Political Science, London, UK; London School of Hygiene and Tropical Medicine, London, UK

**Keywords:** Infected Blood Inquiry, Hillsborough Disaster, Windrush Scandal, Post Office Horizon IT Scandal, statutory failure

## Abstract

**Background:**

Several independent reviews in the UK have recently investigated sustained, systemic failings by statutory services and government departments. These reviews document severe psychological impacts on people affected by these scandals, which have been exacerbated by miscarriages of justice, denials of accountability and lack of formal support. There is evidence that impacted people have significant, unmet mental health needs.

**Aims:**

To explore the psychological support needs of people infected and affected by the infected blood scandal in England, their experiences of seeking support and how insights from this research could inform responses to people affected by similar failings, including the Hillsborough Disaster, Windrush Scandal and Post Office Horizon IT Scandal.

**Method:**

We used a qualitative design involving semi-structured interviews with infected and affected people in England and with mental health practitioners to explore experiences, psychological impacts and perspectives on existing support services. Our analysis was thematic, adopting an empathetic interpretive orientation toward participants’ experiences.

**Results:**

We identified significant unmet mental health needs among infected and affected people, including those who had been in contact with NHS or private psychological support services. Historically, infected and affected people have rarely accessed effective mental health support.

**Conclusions:**

Insights from the Infected Blood Inquiry and the subsequent development of a bespoke psychological support service could inform the setting up of skilled, tailored psychological support for people affected by other severe, systemic state failings. This response could address complex, unmet mental health needs and increase understanding of the psychological impacts of scandals resulting from systemic, statutory failings.

In the UK, several severe, systemic failings by statutory services and government departments have recently been subject to independent review. These reviews have documented severe psychological impacts on affected people, which have been exacerbated by prolonged miscarriages of justice, denials of accountability and a lack of formal support. In addition to calling for transparency about the causes of these failings and adequate financial compensation for impacted people, these reviews have emphasised the need to address significant, resulting unmet mental health needs.

Relevant reviews include (Box 1) the Hillsborough Independent Panel (2012) and the Jones (2017) Reports, which scrutinised the South Yorkshire Police’s responsibility for, and response to, a fatal crowd crush during a football match in 1989. The independent review of the Windrush Scandal (2020) concerned the denial of the legal right to remain as Commonwealth-born UK residents (many of Afro-Caribbean descent) whose entitlements to residency had been lost or destroyed by the Home Office. The Infected Blood Inquiry (2024) addressed the circumstances in which men, women and children treated by the National Health Service (NHS) were given HIV and hepatitis-infected blood and blood products in the 1970s and 1980s, and the subsequent response of government and the NHS to infected and affected people. Currently, the Post Office Horizon IT Inquiry is investigating the responses of the UK’s Post Office, the software company Futjitsu and the UK Government to emerging evidence of wrongful prosecutions of sub-postmasters and sub-postmistresses (self-employed people running branches of the Post Office) for false accounting and theft resulting from faults in Futjitsu’s Horizon IT system.

Box 1 Sustained failings by statutory services subject to recent review in the UK

The **Hillsborough Disaster** resulted in 97 people dying (including 38 children and adolescents) and 766 being injured after South Yorkshire Police (SYP) allowed too many Liverpool football supporters into two fenced ‘pens’ in Sheffield Wednesday’s Hillsborough football ground on 15 April 1989. The SYP and Hillsborough stewards failed to alleviate the crush and, together with the ambulance service, subsequently mishandled the major incident plan due to failures in leadership, coordination and equipment. Although the subsequent Taylor Report (1990) highlighted SYP failings, the aftermath of the Hillsborough Disaster was characterised by a police cover-up, false allegations about drunkenness and hooliganism among Liverpool fans, and a failure to bring criminal prosecutions. It was only in 2016, following the Hillsborough Independent Panel (2012), that a new inquest found all of the victims to have been ‘unlawfully killed’. There have been no criminal convictions in relation to the Hillsborough Disaster.

The **Windrush Scandal** was caused by the failure of the Home Office to preserve records of Commonwealth citizens being granted indefinite leave to remain in the UK under the 1971 Immigration Act, and government policies in the 2010s creating a ‘hostile environment’ for people lacking documentation evidencing their legal status as UK residents. The Windrush Scandal affected at least 13 000 people who had been born British subjects in the Commonwealth and had arrived in the UK before 1973. Many lost their housing, employment and access to healthcare. Some were wrongly detained, denied legal rights and threatened with deportation, wrongly deported from the UK or denied re-entry after travelling abroad. The scandal affected people with Caribbean, South Asian and African heritage, many of whom had begun living in the UK as children, and their families.

The **Post Office Horizon IT Scandal** impacted many people running local branches of Post Office Counters Limited (‘the Post Office’), a state-owned retail post office company, and their employees. Hundreds were wrongfully prosecuted and convicted of theft, fraud and false accounting following the Post Office’s installation of software (‘Horizon’) provided by the company Fujitsu in 1999. Successive versions of this software regularly showed shortfalls in branch accounts, for which the Post Office held branch managers and staff personally responsible. Managers and staff were also prosecuted but not convicted, coerced by the Post Office into making up substantial shortfalls with their own money or had their contracts terminated. The Post Office IT Inquiry has concluded that senior staff at the Post Office ‘knew or, at the very least, should have known that […] Horizon was capable of error’^5^ and that at least 13 people affected by the scandal have taken their own lives and at least 59 have contemplated suicide.

To date, the Infected Blood Inquiry has made the strongest recommendations related to the psychological impacts of sustained, systemic failings by statutory services and government in calling for the establishment of ‘a bespoke psychological service’^1^ to address the unmet mental health needs of infected and affected people.

## Mental health impacts of state-caused scandals

There is little published research on the psychological impacts of systemic failings by state institutions and how these impacts are aggravated by prolonged institutional denial,^2^ although the Jones Report on the experiences of the Hillsborough families,^3^ the Williams review into the events leading up to the Windrush Scandal,^4^ the Post Office Horizon IT Inquiry^5^ and the Infected Blood Inquiry^6^ have all collated evidence of such impacts. There is also a dearth of knowledge about how (NHS and private) psychological and counselling services have responded to affected people’s mental health needs.

This article seeks to help fill this gap. We draw on data collected as part of a study that sought to assess the need, and to make recommendations, for psychological support for individuals and their families in England affected by NHS-supplied infected blood and blood products.^7^ Commissioned by the Department of Health and Social Care in 2022, the study sought to capture the self-reported psychological needs and lived experiences of people infected and affected by the infected blood scandal, including their contacts with mental health services.

In our discussion, we consider parallels between the findings from our study and those from independent reviews and inquiries into the Hillsborough Disaster, Windrush Scandal and Post Office Horizon IT Scandal to inform responses to the unmet mental health needs of people impacted by sustained, systemic statutory failings.

## The Infected Blood Scandal

The Infected Blood Inquiry was established in 2017 as an independent, statutory public inquiry into events that have become known as the infected blood scandal, which has been described as the ‘biggest treatment disaster in the UK National Health Service history’.^6^ Between 1970 and 1991, an estimated 30 000 to 33 000 people in the UK were infected with HIV, hepatitis C and hepatitis B through NHS-supplied blood and blood products, including around 4700 people living with bleeding disorders.^8^ The blood and blood products were supplied from pooled donations (mostly from ‘high-risk’ donors in the USA) despite increasing evidence that they carried significant risks. Infections may have continued beyond 1991.

Many people were co-infected with HIV and hepatitis C or/and hepatitis B. Some infected people passed HIV infection on to their partners. An estimated 2900 infected people died during 1970–2019 as a direct consequence of infection, and fewer than 250 of the 1250 people known to have been infected with HIV (including 380 children) were still alive by 2020.^8^ There are additionally many affected people, including bereaved partners, parents and children, who shared infected peoples’ experiences of the ‘corrosive effects of infection, the debilitating side effects of treatment […], brutal manifestations of stigma […], losses of career, educational opportunity, and finance’,^1^ often involving caring for infected people suffering severe illness.^9^

Two initial inquiries into the infected blood scandal failed to fully investigate its extent, causes or impacts. The 2009 Archer Independent Inquiry^10^ and the 2015 Scottish Public Inquiry into Hepatitis C/HIV-acquired infection (Penrose Inquiry)^11^ had limited access to records and did not apportion blame for the scandal, although the Archer Inquiry recommended access to counselling for infected people.^10^ In July 2017, over 400 infected and affected people brought a group legal action against the UK Government, seeking compensation for infected haemophiliacs and their families.^12^ Later that month, then Prime Minister Theresa May announced the establishment of what became the Infected Blood Inquiry.^13^

The list of issues investigated by the Inquiry included how much the UK Government, blood services other NHS services and medical practitioners knew about the risks of infection, whether sufficient and adequate information was provided to patients and families, and if there was a ‘cover-up’.^14^ The Inquiry’s seven-volume final report, published in May 2024, documents ‘that wrongs were done on individual, collective and systemic levels’^6^ on a ‘horrifying’ scale,^6^ including children living with haemophilia given high-risk blood products ‘as objects for research’ without parental consent,^6^ and through ‘deliberate destruction of some [Government] documents and the loss of others’.^6^

The result of the scandal has been ‘a level of suffering which it is difficult to understand … compounded by the reaction of the government, NHS bodies, other public bodies, the medical professions and others’.^6^ The Inquiry found that infected and affected people have lived with serious long-term mental, physical, social and economic impacts, while spending decades being denied recognition, refused adequate compensation and ‘told that all had been done as well as it could have been when they had reason to believe it had not’.^1^ Drawing on expertise commissioned from its Psychosocial Expert Panel,^15^ the Inquiry described the psychological impact of the infected blood scandal and its aftermath as ‘significant’^9^, ‘profound’^9^, ‘devastating’^9^ and a ‘heavy burden’^9^ on infected and affected people resulting in mental health difficulties for many survivors. On publication of the Inquiry’s report, the UK Government issued a formal apology for these events.^16^

## Method

This study used a qualitative design, involving semi-structured interviews with infected and affected people, mental health practitioners, and NHS services and third-sector organisations working with infected and affected people in England (please see the COREQ checklist in the Supplementary Material available at https://doi.org/10.1192/bjo.2025.10901).^7^

### Public involvement

We consulted the British Red Cross, Haemophilia Society, Haemophilia & Bleeding Disorders Counselling Association, Hepatitis C Trust and Terrence Higgins Trust at the outset of the study. These organisations provided feedback on our research materials (information sheet, interview topic guide), which we incorporated, and facilitated the recruitment of infected and affected people to the research.

### Recruitment

We invited infected and affected people to be interviewed via two routes. First, the Hepatitis C Trust, Terrence Higgins Trust and British Red Cross advertised our study to their subscribers, or through word of mouth, providing an email address for individuals to contact the research team: 36 infected and affected people made initial contact, resulting in 24 interviews. We also had access to contact details of respondents to the 2022–23 Service Satisfaction Survey of infected people and their dependents who are registered with the England Infected Blood Support Scheme (EIBSS) who had expressed interest in participating. We purposively sampled 63 of these respondents to invite for interview (based on age, ethnicity, gender, location and historical contact with mental health support), resulting in 28 interviews (Table 1).

**Table 1:** Sampling of interview participants recruited through the England Infected Blood Support Scheme survey

Interview participant	Invited	Interviewed
Has received discretionary payment^a^ and has had psychological treatment in the past	9	6
Aware of, but has not received, discretionary payment^a^; has had psychological treatment in the past	7	4
Not aware of the discretionary payment^a^; has had psychological treatment in the past	8	3
Has not had psychological treatment in the past and does not wish to access treatment^b^	7	4
Has not had psychological treatment in the past and does not know whether they wish to access treatment^b^	7	3
Has not had psychological treatment in the past and wishes to access treatment^b^	16	5
Did not wish to disclose survey responses to the research team	9	3
**Total**	63	28

a Infected and affected people registered with the England Infected Blood Support Scheme can apply to receive a discretionary payment toward counselling and talking therapy costs for private treatments.

b In response to the question ‘Would you or a family member want to have access to any psychological treatment, support or counselling linked to your, or your partner’s, infection?’ (No; Don’t know; Yes).

People who expressed an interest in being interviewed were emailed an information sheet, giving them the option of an online, telephone or in-person interview, and they were asked for full written consent before the interview was arranged. In total we interviewed 52 people (infected people *n* = 41, affected people *n* = 11; Table 2).

**Table 2:** Demographic characteristics of infected and affected people interviewed

Status	*n*
Infected	41
Affected	11
Bereaved	10
Gender	
Male	20
Female	32
Age group, years	
21–30	1
31–40	3
41–50	8
51–60	17
61–70	13
≥71	10
Recruitment route	
EIBSS survey	28
Other^a^	24
Ethnicity	
Black/Black British, African	1
Asian/Asian British	4
Mixed: White and Asian	1
Mixed: White Other	1
White: British	37
White: Irish	1
White: European	2
White: Other	3
British	2
Region of residence (current)	
East of England	7
London	7
South-East	12
South-West	7
Midlands	8
North-West	4
Yorkshire/Humber	5
Wales	1
Europe	1

a People volunteering for interview after receiving information about the study (via other organisations or word of mouth) including *n* = 3 recruited from the England Infected Blood Support Scheme (EIBSS) Survey.

We additionally interviewed 14 mental health practitioners and experts (13 interviews). The majority of these participants were practising clinical psychologists or psychotherapists (*n* = 10), including in specialist psychological services for infected and affected people in Scotland and Wales. The remainder held service support roles for people with HIV or hepatitis C.

### Data collection

Interviews with infected and affected people explored experiences of the infected blood scandal, impacts on well-being and views on, and experiences of, counselling, psychological and other support. Interviews with professionals focused on their perspectives on existing psychological support for infected and affected people in England. Interview topic guides are included in the Supplementary Material.

### Analysis

The analysis primarily followed the structure of reflexive thematic analysis, supplemented by techniques from grounded theory, including constant comparison and analysis of deviant cases.^17^ The team met regularly and took an open, exploratory, flexible and iterative approach^18^ to collectively develop themes that described the ‘essence’ or core concepts of our analysis.^19^ This process began with initial (open) coding of experiences/events/encounters (through discussion that fed into coding with NVivo (version 12 for Windows; Lumivero, Burlington, Massachusetts, USA; https://qsr-nvivo.software.informer.com/12.2/)) and progressed to axial coding of relationships between initial codes (further developed in NVivo) to describe the ‘essence’ or core concepts of our analysis.^19^ We adopted an empathetic interpretive orientation toward participants’ experiences.^20^

### Reflexivity statement

The study team consisted of five academic researchers with no previous personal or research experience of the infected blood scandal. The team did not include clinicians, but contained extensive experience of working in mental health support and conducting research in health and social care, including on sensitive topics. We collected, analysed and reported on our interview data before the Infected Blood Inquiry published its final report, familiarising ourselves with the infected blood scandal by consulting organisations providing support to infected and affected people, reading and listening to evidence from the Inquiry and reading the Inquiry’s interim reports.

The experience of interviewing was complex for the researchers who spoke to infected and affected people (J.C., E.C.) and mental health practitioners and experts (E.N.). The teams’ desk-based research had not fully prepared us for the complexity and duration of people’s experiences of the infected blood scandal (particularly their interactions with health services) or for the intensity of our own emotional and mental responses to interview participants’ accounts, which included confusion, outrage and distress about what had happened to infected and affected people.

Throughout the research process, we were mindful of the potential for our reactions and perspectives to influence both the interviews and our data interpretation. We made a conscious effort to set aside our personal responses, which we discussed in team meetings. Our aim was to ensure that our analysis and recommendations were grounded in the experiences that infected and affected people shared with us and situated in wider knowledge about the infected blood scandal, particularly the work of the Infected Blood Inquiry and specialist support organisations.

### Ethics statement

The authors assert that all procedures contributing to this work comply with the ethical standards of the relevant national and institutional committees on human experimentation and with the Helsinki Declaration of 1975, as revised in 2013. All procedures involving human participants were approved by the Observational/Interventions Research Ethics Committee at the London School of Hygiene & Tropical Medicine (reference number 28215). Written and verbal consent was given before interviews. Participants were able to withdraw from interviews and had the opportunity to review their interview transcripts.

## Results

Infected and affected people reported five main psychological impacts of the infected blood scandal. Grief and loss included infected people’s regrets about the impact of prolonged illness, depression and treatment on their relationships with their children, and affected people’s estrangement from their infected parents. Anger was particularly focussed on the lack of accountability for NHS use of infected blood and blood products, and subsequent failures to provide redress or support. Fear and anxiety were related to issues including financial security for dependents and infected people’s future health. People also described experiencing guilt resulting from situations such as unknowingly administering infected blood products to their children, or because of the stress their illness caused their families. Additionally, people reported feelings of stigma and/or isolation. Although only about half the participants explicitly identified themselves as experiencing trauma, all described incidents that had caused them significant distress.

Participants broadly described four periods in their lives, which sometimes overlapped, during which their mental health support needs were not met: (a) at the point of diagnosis and during related treatment; (b) while coping with impacts of infection on multiple aspects of their lives; (c) when seeking psychological support and (d) during the Infected Blood Inquiry itself. Experiences described by infected and affected people span four decades, from the mid-1980s to the mid-2020s, often with simultaneous stresses on their psychological well-being. Many people described coping with infection (or infection of a loved one) in terms of trying to get on with their lives, but all could recall times when they would have benefitted from mental health support.

In the following, we report on the four life stages described above. Study participants are identified by number. Numbers starting with ‘1’ refer to infected people, those with ‘2’ refer to affected people and those with ‘3’ refer to mental health professionals.

### Adapting to diagnosis and treatment

Almost all participants described profound failures in how they were informed about infection lacking ‘a proper mechanism*’* [122] or an offer of support after diagnosis.

Most infected participants recalled being informed about their infection in an *ad hoc*, ‘very matter of fact’ [111] way, including as children, in inappropriate venues, such as a hospital corridor or waiting room, in passing by unfamiliar medical staff (‘Oh, you’re the kid with hep C’ [117]) or as it ‘sort of slipped out’ [122] during routine medical appointments. One person infected with HIV as a child remembered being spoken to ‘like I was an adult. And they were so cold; there was no compassion, there was no care and certainly no thought of psychological welfare or support or anything’ [110].

People living with haemophilia as children in the 1980s described an emerging awareness of hepatitis C and HIV, often based on ‘a lot of misunderstanding, a lot of assumption because of the lack of information’ [135]. One participant, co-infected with HIV and hepatitis C, described the uncertainty as: ‘“You may have flu or you may die.” But then, more and more people kept dying and less people seemed to have flu so there was a shift. But you weren’t really told; you had to learn this for yourself’ [116].

There appeared to be ‘an assumption [in] the medical world that we knew, and that we’d been catered for… where they’d told us about this illness and that they’d supported us, which of course they hadn’t’ [122]. Some parents tried to shield their children from knowledge of their own or their children’s infection which, in the absence of follow-up from the NHS, could have serious long-term implications. One participant, whose father died as the result of infection, did not know about his own infection until adulthood, although it was recorded in his childhood medical records, and he suspected his parents had been informed [111].

People infected through blood transfusion, without access to knowledge shared in the haemophilia community, were sometimes informed following unexpected hospital ‘call backs’. However, most endured years of ill health before they were given blood tests identifying infection, often struggling to have symptoms taken seriously. Several were asked intrusive questions about their alcohol and drug use and sexual history when diagnosed.

Many people remembered being given some information about their prognosis and advice (some of which is now outdated) on infection control, such as guidance to always use a ‘separate bathroom’ [135]. A few recalled being strongly advised to keep their status ‘secret’ [102], including from siblings [135], resulting in living in ‘a bubble of secrecy’ [102]. Several described HIV and hepatitis C infection as an acutely ‘stigmatised thing at that time’ [102].

Treatment of infection was described as gruelling, with severe impacts on capacity to work and maintain family life, and side-effects including chronic fatigue and mood swings, causing ‘the trouble, the upset, the trauma’ [105] of disrupted family life during which children moved out, went into foster care [113] or dropped out of school. Some people reflected that even completing ‘successful’ treatment had lasting effects: ‘I’ve never wanted a career. I’ve not adapted to social norms because that wasn’t how my life was projected. I wasn’t going to have a career, because what’s the point of having a career when you’re going to be dead’ [135].

Not one of the people we interviewed was offered counselling or psychological support at point of diagnosis. Only one person remembered being offered counselling during treatment for hepatitis C. Another described not being offered support during treatment as late as the 2010s: ‘There was nothing. I went in every two or three weeks, they were, “Go and take blood.” And it was go in, take blood, get a prescription, go to the pharmacy, get the treatment, go home. That was it. There was no, not even a “How are you feeling?’” [108]. This lack of support or accountability was reported by many infected and affected people as compounding the impacts on their mental health: ‘[S]ometimes it’s not the actual original trauma that distresses people, it’s how they’re treated afterwards that becomes the trauma’ [306].

#### Coping with the impacts of infection on daily life

Direct impacts of infection included death and bereavement, chronic illness, caring for people during prolonged illness, difficulties making decisions about sexual relationships and pregnancy, stigma and harassment at work and in the community, strain on family relationships, and loss of career and income. Participants were also often unable to separate the toll of dealing with these impacts from their capacity to cope with other stressful life events not directly related to infection.

Many people experienced bereavement. Infected and affected participants described their grief for spouses, partners, parents, siblings, uncles and friends, and living with distressing memories of their ill health, treatment and circumstances of their deaths. Some lasting impacts of these bereavements included guilt, feeling ‘robotic*’* [209], a frequent sense of the loved one’s absence [203] and vivid, painful recollections [212]. Some described how death of a parent (from natural causes) left them without support they had relied upon when coping with their own or a loved one’s infection.

Infected people, including some who self-cleared or received successful treatment for hepatitis C, reported complex health issues, including liver cirrhosis, joint damage, brain fog, cardiovascular disease and cancer, often experienced as multimorbidities compounded by ageing. This severely affected their capacity to work, participate in family life, engage in activities and interact with their communities.

Diagnoses of infection altered decisions about sexual relationships and family planning, complicated by considerations of infection risk and life expectancy. Participants described the ‘brutal negligence’ of having ‘no counselling, …no support’ in understanding, and adjusting to, infection within intimate partners [101]. A few participants remembered medical advice to terminate pregnancies in the 1980s, and several described relationships ending or couples deciding not to have children. Many infected people with children worried about the lasting psychological impact of their infection on their children into adulthood.

Infected and affected people often experienced stigma or harassment, including feeling isolated socially or at work, being bullied and isolated at school, having their home vandalised, being subject to assumptions in the health system that infection was the result of drug use or sexual activity, and feeling marked out as an infection risk in medical settings.

Although individuals may have encountered empathetic, informed allies (in their general practitioner, haemophilia nurse, etc.), there was broad consensus across interviews that people have *‘*been massively neglected over a very long period of time’ [306].

#### Dealing with mental health difficulties or crises

Infected and affected participants who had sought formal therapeutic support in England had been unable able to find services that understood the impact of the infected blood scandal. Some recalled long periods of hopelessness when they disengaged from future-orientated plans, lost jobs, withdrew from family and friends, or self-medicated with alcohol or drugs. Several experienced ‘breakdowns*’* [116], not wanting to live [206] or depression. Some also talked about currently or recently trying to manage with debilitating psychological states including ‘flashbacks*’* [206], feelings of being ‘worthless*’* [141] and intrusive thoughts [212]. A few described years of just about managing to cope with the psychological impacts of infection. Most interviewees had not sought psychological and counselling support. Practitioners explained this as the result of a ‘fear of re-traumatisation’ [303], a legacy of distrust in institutions [307], lack of knowledge about services [311] and restrictive criteria for accessing them [305].

A significant minority did describe trying to access or accessing NHS or private therapies. Some had received one-to-one and group support from staff at third-sector organisations working with different groups of infected people, which they valued as empathetic, informed, responsive and adaptive to their needs. Very few accessed an offer of discretionary funding from the EIBSS for private therapy, which was increased during the period of our research to £900 per annum (with the option to apply for further treatment funding). Therapies accessed ranged from bereavement counselling, psychoanalytic psychotherapy, sessions with NHS talking therapies (psychological therapies delivered by trained and accredited NHS practitioners) and eye movement desensitisation and reprocessing.

Those who accessed treatment reported multiple difficulties navigating the complexities and limitations of seeking psychological support. Some reported good experiences with ‘very gentle, very positive, very listening’ [106] and ‘brilliant’ private therapists or a ‘multi-professional, multi-dimensional’ NHS-based team [137]. However, receiving appropriate, informed support was unusual and generally by chance.

People referred by hospital clinics or general practitioners for NHS support were usually referred onto waiting lists for ‘time-locked’, solution-focused [117] sessions that then ‘just touch the sides’ [114]. NHS practitioners confirmed that there is a lack of robust evidence on how to best address the needs of infected and affected people: ‘There is very little research about this population now, so I guess any guidelines […], first of all, need to work from acknowledging this’ [312].

People who sought support from private practitioners had to *‘*randomly search for a psychologist’ or counsellor [303], but did not receive any guidance about choosing a suitable therapist: ‘You don’t know whether you need counselling, whether you need psychological support, whether you’ve got [post-traumatic stress disorder]. You don’t know whether it’s anxiety, whether it’s generalised anxiety, whether it is depression. You’ve got to almost quantify it yourself’ [303].

Participants identified the overriding obstacle to receiving suitable support (NHS or private) as a general lack of knowledge about the infected blood scandal: infected and affected people were forced to repeatedly explain ‘very difficult’ [123], complex and unfamiliar experiences to practitioners, who sometimes responded inappropriately. Several participants described situations where they were advised to ‘put [the scandal] in a box and put it to one side’ [131]. One recalled that ‘[the counsellor] actually turned around to me and said, “Oh my gosh, I’m getting stressed listening to it. I don’t know how you cope”’ [104].

Infected people living with severe mental illness described having their experiences of the infected blood scandal downplayed in their interactions with the mental health system: ‘I think what they’re doing is they’re compartmentalising things. That’s bi-polar, she’s depressed because she’s had hepatitis C and the treatment has left her whatever. We’ll send her here because she’s frightened, they’re not lumping it together, the whole thing has caused all of this’ [141].

#### Involvement with the Infected Blood Inquiry

Participants’ involvement in the Inquiry spanned a spectrum from being aware of some of the proceedings to regularly attending the Inquiry and submitting evidence. Many participants were initially sceptical about the Inquiry, after years of campaigning or waiting for justice, but had been won over by the Inquiry’s Chair and his conduct during sessions, which inspired confidence in the expected final report.

The Inquiry was described as having been ‘cathartic*’* [122] and a source of solidarity and community for many infected and affected people [123]. However, it was also a cause of distress, both increasing awareness of, and vulnerabilities around, the infected blood scandal [108]: ‘There are people who have listened every day to the Inquiry and have heard the most horrific, heart-breaking stories. There are people who’ve lost friends, lost family members during this Inquiry […] People are exhausted, they are spent, they’re absolutely spent’ [310].

One professional summarised the experience for many of the Inquiry as a culmination of events initiated by ‘the infected blood situation, then you have how it was handled, and then you have years of having to fight to be heard and to have your experience heard, and for somebody to take account of that’ [306].

The Red Cross was appointed by the Inquiry to provide listening/counselling service to attendees, and encountered significant unmet psychological needs. Participants in contact with the Red Cross – many of whom had not previously sought psychological or counselling support – valued speaking to someone empathetic [105, 121] with a detailed understanding of the infected blood scandal and the Inquiry [113], who frequently remembered them from previous contact. Participants ascribed a range of positive qualities to the Red Cross counsellors, including that they really listened [105], that they empowered infected and affected people [107], provided ‘a sort of first aid, safe place to cry’ [114], and enabled people ‘to share their story and be appreciated and acknowledged’ [123]. However, for some participants talking to this ‘brilliant’ [114] service, although ‘helpful’ [104], did not feel adequate for their needs. Moreover, not everyone was aware of this support.

### Discussion

This research has documented a lack of adequate response to the serious, ongoing mental health needs of infected and affected people related to severe and prolonged, systemic failings of the UK Government and the NHS.

The Infected Blood Inquiry raised concerns early on about the lack access to psychological support for infected and affected people,^21^ which the devolved administrations in Northern Ireland (2019) and Wales (2020) responded to by setting up specialised services. Scotland had already begun introducing psychological support services for people with inherited bleeding disorders following the 2015 Penrose Inquiry, with the Scottish Infected Blood Psychology Service established in 2021 to support anyone infected or affected by infected blood.^7^

In England, in recognition that the existing psychological support services had not been meeting the needs of the infected and affected communities,^22^ a bespoke psychological support service was commissioned in 2023 and launched in June 2025.^23^ The England Infected Blood Psychological Service covers infected and affected communities across England, offering ‘comprehensive assessment, support and treatment to improve the psychological health and wellbeing of anyone who has been infected or affected by the infected blood scandal’.^23^ The Service broadly aligns with the several attributes that our research identified to be important for a psychological support service (Box 2).^7^

Box 2 Key attributes of a psychological support service for infected and affected people

Infected and affected people with a range of experiences should be involved in the development and design of the psychological support service if it is to be effective in supporting these diverse communities.

Practitioners providing the service should be adequately qualified, accredited and registered, and have:

- an interest in and experience of working with trauma-affected populations;
- experience of working with people who have long-term health conditions that affect mental health and *vice versa*;
- be self-motivated and proactive in terms of willingness to reach out and making themselves available;
- be flexible in the way they work;
- and, most importantly, have sensitivity to, and knowledge about the infected blood scandal, and related health conditions.

The service should work to establish trust, including through outreach with attention to people less able to engage with services.

It should be compassionate, respectful of its clients and non-judgemental.

It should be accessible through various routes, including self-referral; available to a wide circle of infected and affected people; and able to respond to needs resulting from diverse, individual experiences.

It should be flexible and agile, offering the ability for infected and affected people to access the service when they need it and to re-enter it, employing a range of modes (in person, online, telephone) and therapeutic modalities (including advice on alternative therapies and social prescribing).^7^

The psychological needs of people affected by the Hillsborough Disaster, the Windrush Scandal and the Post Office Horizon IT Scandal, including family members, have not been as fully investigated as those of people affected by the infected blood scandal. Neither have people affected by these other scandals been formally provided specialised psychological support. However, serious and complex mental health needs have been identified in independent reviews of all three scandals, which, in common with the infected blood scandal, involved severe and sustained failings by statutory services and government departments, followed by prolonged miscarriages of justice, including denials of accountability and a lack of support.

The 2017 Government-commissioned Jones Report into the Hillsborough Disaster found that ‘the disaster, the aftermath, and the struggle to be heard … had an adverse effect on the mental and physical wellbeing of both families and survivors. Depression, marital breakdown, family division, mental illness, unemployment, premature death and even suicide have featured in the Hillsborough narrative’.^3^ It recommended ‘support and counselling in the aftermath of a public tragedy’.^3^ In 2021, the South Yorkshire Police, together with the West Midlands Police, in acknowledgement of their errors during investigations, agreed to pay compensation to bereaved family members and damages to survivors, with access to a fund for psychiatric treatment or counselling.^24^

The 2020 Williams Review of the Windrush Scandal stated that the scandal had been both ‘foreseeable and avoidable’, and criticised ‘a culture of disbelief and carelessness’ in the Home Office.^4^ The Review found that people had lost their jobs and homes; been denied access to benefits, healthcare and pensions; and experienced homelessness in the UK and destitution following deportation. Many of their relatives, particularly children, were also affected. The Review reported feelings of injustice and betrayal and impacts on people’s mental health related to stress, increased alcohol consumption and social withdrawal. It recommended that the Home Office ‘should agree to work with other departments to identify follow-up support, in addition to financial compensation’.^4^

The Post Office Horizon IT Inquiry’s 2023 interim report, based on human impact hearings with 189 post office branch managers, assistants and family members, found that ‘the scale of the suffering and financial loss which so many have endured [… indicates] a compelling need to provide compensation to all those who had suffered loss and damage which properly reflected their pecuniary and non-pecuniary losses’.^5^ The Inquiry subsequently reported that many impacted people ‘suffered psychiatric or psychological harm … [which] was a very likely consequence of investigation, prosecution, conviction and sentence.’^25^ Research with 101 branch managers or their employees who were wrongly accused, convicted or investigated suggests that they are living with significant post-traumatic stress (67%) and depressive (60%) symptoms irrespective of the outcome of their case.^2^

These reports document that the Hillsborough Disaster, the Windrush Scandal and the Post Office Horizon IT Scandal have had similar mental health impacts to those found by the Infected Blood Inquiry. These impacts have been aggravated by experiences of being disbelieved, stigmatised or socially excluded, which has undermined trust in institutions and belief that justice can be achieved.^4,7^

Our research suggests that people impacted by each of these systemic, sustained failings could benefit from accessible, trustworthy, skilled, tailored psychological support services rooted in knowledge of the specific scandal and its impacts. Delays in responding to the psychological impacts of these sustained, systemic failings have compounded unmet mental health needs,^3–6^ suggesting that scoping should be made as early as possible to minimise ongoing impacts and reduce distrust. Scoping and planning for each potential service should involve a diversity of affected people and relevant community and mental health experts to increase knowledge about their mental health needs, the barriers to them accessing psychological support (including distrust and negative experiences of mental health services), and their preferences for therapeutic modalities and modes of support.

In conclusion, the Infected Blood Inquiry, in common with investigations into the Hillsborough Disaster, the Windrush Scandal and the Post Office Scandal, has strongly indicated that these scandals have left affected people and their families, with serious and complex unmet mental health needs. The provision of specific psychological support for infected and affected people in England following the infected blood scandal suggests that other groups affected by scandals resulting from systemic, sustained failings by statutory services and government could also benefit from bespoke, co-designed services to meet their specific psychological needs.

#### Strengths and limitations

Research participants were recruited through support organisations and a survey of those registered with the EIBSS, potentially excluding individuals who were not engaged with these services. We interviewed a larger number of women and people identifying as White British than reflects the UK population. The former supports evidence that men are more difficult to recruit to health studies than women, especially in qualitative research.^26^ The latter may indicate something about the demographics of the current infected and affected community, but we do not have data about the total infected and affected population, nor on marginalisation from services (including the EIBSS) within this population.

## Supporting information

Carlisle et al. supplementary material 1 Carlisle et al. supplementary material

Carlisle et al. supplementary material 2 Carlisle et al. supplementary material

## Data Availability

Interview transcripts for this study are not publicly available. Research participants were not asked to consent to their data being shared due to the extreme sensitivity of this research and potential risks to their privacy.

## Supplementary material

The supplementary material is available online at https://doi.org/10.1192/bjo.2025.10901
