# Supplementary material for: Psychological support for people affected by scandals caused by serious and sustained failings of statutory services and government: lessons from the infected blood scandal and Infected Blood Inquiry
Source: BJPsych Open. 2025 Nov 25;11(6):e286. doi: 10.1192/bjo.2025.10901 (PMC12724117; doi:10.1192/bjo.2025.10901)
Supplement: Carlisle et al. supplementary material 1 — Carlisle et al. supplementary material [file S2056472425109010sup001.docx]

**Supplementary File 1: Interview guides**

# **Interview Guide A: Infected and affected people**

We want to find out your views on counselling and psychological support and any experiences you have had of using, or thinking about using, this kind of support. The aim of the project is to understand what the support should look like, and to help improve future services for people infected or affected by contaminated blood or blood products.

We believe it is important to ask people who have been directly affected by this tragedy what they think [about psych support services]. We are grateful that you have agreed to talk to us.

Everything you say is confidential and anonymous. I will record the interview, but the recording will be deleted once it has been transcribed (this will not include your name or any identifiable information). You can review the text/transcription, if you like. Is that okay?

[First, let people talk about their views and their experience. If possible, discuss the questions below as part of a conversation].

- Can you tell me a little bit about yourself and your experiences of the contaminated blood scandal/tragedy?
- Are you in touch with any charities or networks that provide information and advice to people who have been infected or affected by NHS-contaminated blood or blood products? If so, how have they been helpful to you?
- Do you receive information or advice from anywhere else (haemophilia clinic, peer-support group etc.)?
- What has been the impact of the contaminated blood tragedy/scandal on your own and your family’s/loved ones’ mental and emotional wellbeing?
- I have some questions about your views and experience of counselling, psychological and other types of support for your well-being.
- Have you accessed any professional support or help with your emotional or mental wellbeing?

If yes: Why did you decide to seek this support (what was happening in your life, how were you feeling)? [NOTE: may have accessed support more than once.]

- - How did you find out about it and then accessed/arranged it?
  - Who was the provider/therapist? (Red Cross listening ear service through the Public Inquiry, NHS, charity, HBDCA, private) and what were your reasons for choosing them?
  - Can you tell me a bit about the support/sessions? Were they helpful?
  - What would have made it better (type of support, length of support, expertise of therapist, ease of access and form of delivery, e.g. in person or online)?

If no: What information have you had about support?

- - Have you ever considered accessing support? If so, what was happening/how were you feeling around the time?
  - What types of support did you think was available?
  - What did you think the pros and cons would be of getting support?
  - What prevented you from getting support (felt able to cope without it, didn’t feel it was for you)?
- Do you know about the England Infected Blood Support Scheme? Are you registered with the Scheme?
- Have you heard about the counselling and talking therapy funding through the Scheme?

If yes:

- - How did you hear about it?
  - Have you applied for it/received it?
  - Was this a one-off payment or have you applied more than once?
  - What support have you been able to access with this funding?
  - What was the result? Was it appropriate/effective/sufficient?

.. questions about what the service should look like.

- What kind of support service do you think would help you or other people?
- Would it be useful to have access to someone who understands the context (e.g. ‘broker’, hub) and who could help you find the appropriate support?
- Who/which type of organisation is best placed/most trusted to provide support services?
- How far would you be prepared to travel to a bespoke service?
- Would it need to be face-to-face or would you consider video or phone consultation?
  - Do you have any experience of video or phone consultations or therapy?
- How important is it that the therapist understands the contaminated blood scandal?
- Do you think continued support would be useful, (e.g. there should be no limit on how may sessions of therapy people are offered)?

Demographic questions

To finish off, I just need to ask a few questions to find out some background details if that’s okay.

[If it is not clear by now, ask about their condition(s), if they were infected or co-infected and which virus, or affected, e.g. spouse, family member etc.]

| What region do you live in? | | |
| --- | --- | --- |
| How would you describe your ethnicity? | White British  Irish  Other White background  White and Black Caribbean  White and Black African  White and Asian  Other mixed background  Indian | Pakistani  Bangladeshi  Other Asian background  Caribbean  African  Other Black background  Chinese  Other ethnic group |
| Age bracket? | 18-30 / 31-40 / 41-50 / 51-60 / 61-70 / 71-80 | |
| Gender |  | |

Could I ask you to give me a sense of your psychological state at this point on a scale of 1 to 10 (with 10 being the best state and 1 the worst)?

Is there anything that we have not covered that you think is important to help inform the development of appropriate mental wellbeing support services for infected/affected people?

Thank you very much for your time. It has been really helpful for the project.

# **Interview Guide B: Practitioners & Decisionmakers**

This topic guide is designed to understand your views on counselling and psychological support for people historically infected by NHS-supplied blood and blood products and/or their affected family members and loved ones. We are interested in learning about your experiences of providing and/or overseeing such services, and specifically of setting up/delivering services in the devolved nations of the UK. We are also keen to hear your views of what a future service in England should look like, and what might help infected and affected people to deal with their distress and trauma. Our focus is on counselling and psychological support, but there may be other forms of support that you think can play an important role in improving the wellbeing of infected/affected people.

General questions about counselling and psychological support for infected/affected people

- Could you briefly introduce your role, and describe your area of expertise?
- What is your experience of providing (or overseeing) support to (for) people infected or affected by contaminated blood or blood products?
- How would you describe the current need for counselling and psychological support for infected/affected people in Northern Ireland/Scotland/Wales?
  - Is this likely to change once the Infected Blood Inquiry is concluded and if so in what ways?

Specific questions about psychological support for infected/affected people in Northern Ireland/Scotland/Wales

Northern Ireland, Scotland and Wales have each developed a bespoke psychological support service addressing the specific needs of the infected and affected community.

- How would you describe the current availability and appropriateness of counselling and psychological support services offering for infected/affected people in Northern Ireland/Scotland/Wales?
- Could you describe how the service was set up?
  - Probe: who was involved, how were the needs identified, where was/is the service located (NHS, government, other?), what considerations influenced/shaped the design of the service?
- What were the key issues (positive/negative) that you encountered when setting up the service?
- How did you communicate the setting up/development of the service to the infected/affected community and why?
- What does the service entail and who provides it?
- How were practitioners identified?
  - Probe: what specific competencies do the practitioners who provide the service have or should have?
- Have the practitioners providing the service received any specific training with the infected/affected community in mind and if so, who provided this?
- Could you describe how infected/affected people access the service?
  - Probe: have you had any feedback on how easy do you think do infected/affected people find it to access the service?
- By November 2022, the service in Northern Ireland had served the needs of 38 people [in Scotland had received over 150 referrals] [in Wales has had ~80 referrals]. How well do you think do these numbers reflect the need for psychological support in the infected/affected community in Northern Ireland/Scotland/Wales?
  - Probe: Do you believe these numbers will increase and if so why/how?
- What are the main barriers for infected/affected people to access the service? [skip if already covered above]
- How is the service funded and what are your views on its sustainability?
- In Northern Ireland the support services are linked to haemophilia services for all people with inherited bleeding disorders. What (if any) plans are there to extend the support to people who have been infected/affected through other routes (e.g. blood transfusion)?
  - Probe: what would be the incentives for/barriers to do this?
- In Scotland there are currently two routes to support: (i) embedded with haemophilia services for all people with inherited bleeding disorders and (ii) the Scottish Infected Blood Psychology Service aiming to support those infected through blood transfusion. What (if any) any plans are there to link these services?
  - Probe: what would be the incentives for/barriers to do this?
- In contrast to Northern Ireland and Scotland, Wales provides access to psychological support for all people infected/affected through one routed via the Welsh Infected Blood Support Scheme. What do you think are the advantages and disadvantages of this approach?

Views on psychological support for infected/affected people in England

- In England there is discussion of developing a central ‘resource’ (such as guidelines) to support mental health practitioners providing counselling and psychological support services to infected/affected people. What are your views on the usefulness of such a resource?
- What role do you think could the “Infected Blood Psychology Network” that Northern Ireland, Scotland and Wales have formed play in supporting/informing the set up of a support service in England?
- What do you see as the main challenges of providing counselling and psychological support services to infected/affected people in England?
- What other forms of wellbeing support should be offered to infected/affected people and why?

Close

- Is there anything that we have not covered that you think is important to help inform the development of appropriate mental wellbeing support services for infected/affected people?
